# Dr. Yeats, on Nitrous Fumigation

**Published:** 1800-05

**Authors:** G. D. Yeats

**Affiliations:** Bedford


### *To the Editors of the Medical and Phyſical Journal.*

GENTLEMEN,

I Shall be obliged to you to inſert the incloſed remarks in the next number of your Journal. I remain,

*Bedford,* Your's, &c.
*April* 7, 1800. G. D. YEATS.

---

I intended by my letter to Dr. Duncan, reſpecting nitrous fumigation, to convey an accurate ſtatement of the chemical nature of the gaſes mentioned in the controverſy upon that ſubject. During converſation with medical friends, as well as in publications, I found that ſome inaccuracy prevailed reſpecting their nature and operation. Conceiving that the ſmalleſt error in the premiſes would lead to very falſe concluſions, in which Dr. Trotter had already laid very great ſtreſs upon the deleterious effects of *nitrous gas*, I flattered myſelf that a detail of the chemical agency of azot, azotic gas, nitrous gas, and nitrous vapour, would aſſiſt in throwing ſome light upon the controverſial points. I ſay nothing further, for obvious reaſons, upon Dr. Trotter's letter to Mr. Nepean; but, in the ſecond volume of Medicina Nautica, I find no recantation of error, no confeſſion of ſins. The ſtatement alſo was tranſmitted to Dr. Duncan in February 1799, a whole year before Dr. Trotter's letter appeared in the thirteenth number of the Medical and Phyſical Journal. Merely uſing the words nitrous vapour, conveys no correction of error; it is at beſt but a negative confeſſion, woven gradually into the thread of his narrative, without any direct and manly recantation. In the letter alluded to above,* Dr. Trotter traces fumigation to a ſuperſtitious origin, and throws out a continued ſeries of ridicule againſt that proceſs. From whatever cauſe any valuable art may have taken its riſe, it argues nothing againſt the utility of that art. The ridicule of medical theories, in the diſſertation of Radical Heat and Radical Moiſture, by Uncle Toby and Corporal Trim, forms no objection to the noble attributes of the medical profeſſion; neither, with ſerious men, do the fundamental principles of Chriſtianity receive any injury from the ſuperſtition and vicious knavery of any particular ſect, or the artful ſneers of an eloquent hiſtorian. Such conduct is more

---

* Medical and Phyſical Journal, Numb. XIII. p. 245.

becoming the Cervantic ſpirit of a Swift or a Sterne, whoſe eccentric lucubrations are laid upon the ſhelf for an hour of trifling amuſement, rather than for the exerciſe of ſerious reflection.—"If a philoſopher ſpeaks, men hear him willingly while he keeps to his philoſophy. So is a Chriſtian heard, while he keeps to his profeſſed charity and meekneſs."*

The farce acted before Lord Bothwell and Sir K. Digby, with an infernal agent of his majeſty of darkneſs, is replete with nothing but its own refutation; neither will the fumigations produced from the heterogeneous maſs of tobacco and pine-wood, juniper and tar, and a long liſt of et ceteras, however extenſively they may have been employed, or however generally they may have failed of ſucceſs at New York or at Moſcow, furniſh any arguments againſt the utility of the nitrous proceſs.—I am no advocate for fumigations as formerly employed; they appear more calculated to deceive by enveloping diſagreeable ſmells, than to be beneficial by neutralizing or deſtroying contagious miaſma. But ſtill ſuch a proceſs is not to be haſtily and inconſiderately given up, much leſs is it to yield to the equivocal teſt of ridicule and banter. If a doctrine, which has received the ſanction of 3000 years, is to be laid aſide, let it be done upon firm and ſolid grounds, upon fair and candid experiments, elucidated by a free, manly, and diſpaſſionate inquiry.

Having made theſe remarks, permit me, Gentlemen, to add ſome obſervations in anſwer to Dr. Trotter's critique upon my letter in Dr. Duncan's Annals of Medicine. With reſpect to the ſubject of the fever, I am ſorry I cannot give the Doctor all the information he requires. I attended the patient in November 1798; the weather during that month was bleak, the wind continuing, generally, in north-eaſterly points. He was confined to priſon for poaching game; and in order to obviate the effects of the depreſſing paſſions, I told him, from authority, that he would be liberated upon his recovery. No other perſon ſlept in the room, which is ſmall, but ſufficiently airy, and is upon the ground floor. I could not trace the contagion to its origin; but from whatever cauſe it took its riſe, I pledge myſelf to Dr. Trotter that I made no alteration in the *ſituation* of the priſoner, except ordering clean linen, the dirty to be plunged into water, and the ſtraw, on which his bed-clothes were ſpread, to be burnt. The circumſtances of cleanlineſs and ventilation are *always* rigidly attended to by the gaoler, who is remarked for his humanity to the priſoners. They all,

* Shafteſbury's Characteriſtics, Vol. I. p. 67.

with with one accord, mentioned the agreeable ſenſations they felt after the fumigation was performed.

The *principles* upon which I conducted the nitrous fumigation, are ſtated in my letter to Dr. Duncan, which, I hope, will receive additional elucidation from the ſubſequent parts of theſe obſervations; I leave it to the Public to judge of the utility of the *practice* from experience and accumulated teſtimony.

To diſcuſs the nature of contagion would ſwell the limits of this paper to an improper length; and indeed, on a ſubject ſo intricate, and involved in ſo much obſcurity, I do not wiſh *at preſent* to hazard an opinion. The diſagreeable ſmells certainly favor the idea of the combinations of hydrogen gas with ſulphur, carbon and phoſphorus; but whether theſe, ſeparately or combined, be the contagion itſelf, a diſpoſition of the atmoſphere favorable to its production, or the vehicle of the contagion—adhuc ſub judice lis eſt—and a great benefactor to ſociety will be the perſon who accurately demonſtrates its nature.

That theſe gaſes are generated from putrefactive animal and vegetable ſubſtances, is well known; and that they produce diſagreeable effects upon the human frame is alſo a matter of ſufficient notoriety. Theſe points require no explanation; but it is not quite ſo clear, that oxygen will unite with theſe gaſes at the common temperature of the atmoſphere; and I imagine it requires ſomething more than mere contact to produce a chemical union between them. That oxygen is evolved by the nitric vapour, I can prove to Dr. Trotter from the moſt reſpectable authorities. The Doctor will find it related by Chaptal, that, according to the valuable experiments of Berthollet, the *whiteſt* nitric acid will give out oxygen upon expoſition to the ſun's light;* and in Lavoiſier's Elements of Chemiſtry it is mentioned, that nitric acid has but a ſlight hold of oxygen, and quits it readily to a great number of bodies by the aſſiſtance of a gentle heat.† The fourth degree of the impregnation of azot with oxygen, the ſame illuſtrious chemiſt has termed oxygenated nitric acid;‡ and it is this, I think, which is diſengaged in Dr. Smyth's experiments. After the above ſtatement, and the account I ſent to Dr. Duncan, candour muſt acknowledge the following poſitions to be incorrect: "Dr. Smyth himſelf, we are apt to believe, muſt have been ſtrongly impreſſed with the dread of unfolding the weak ſides of his doctrine, that he ſays nothing of the primitive principles of his nitrous

---

* Elements of Chemiſtry, Vol. I. p. 23. Nicholſon's Tranſlation.

† Kerr's Tranſlation, p. 189.

‡ Table facing p. 185.

gas. That muſt have ſoon reduced him to inexplicable difficulties; for he muſt have ſought its conſtituent parts in the final decompoſition of animal matter, by putrefaction; and after being evolved from his pipkins, whether in ſhips or hoſpitals, from its well-known elective attractions, he muſt have traced it through all its various degrees of oxygenation, till it became nitrous acid, *without imparting a ſingle quality to the air to ſupport life; but on the contrary, to render it ſo much* the worſe, by the quantity it required to bring it to the ſtate of an acid."*

The end in view from the nitrous fumigation, is, no doubt, to render the atmoſphere purer by affording oxygen. Dr. Trotter acknowledges that the nitric fumes neutralize the diſagreeable ſmells by chemical agency; this I imagine does take place, and alſo that the ſuperabundant oxygen is let looſe from the oxygenated vapour. I by no means think that ventilation, and a free circulation of air, are to be neglected; from this conceſſion of his opponents, Dr. Trotter ſeems to triumph over the arguments brought againſt his poſitions. If we can by the powerful aid of chemical agents neutralize diſagreeable exhalations, is it not a deſirable object? Can Dr. Trotter deny that the contagion is not connected with theſe diſagreeable ſmells, and that thus, by decompoſition, we entirely prevent its diffuſion? Is it not probable that ventilation alone, without ſuch aid tends to ſpread the infectious miaſma, particularly if the ſtate of the atmoſphere be favorable to its generation? I apprehend, that many have confounded the nature of contagion with its effects upon the human frame; and in ſearching for an antidote, have ſolely kept in view the protracted ſymptoms of typhus fever. Fumigations may, in this point of view, be ſerviceable in rendering contagious matter inert, although they prove diſagreeable to the ſick; in this way infected wards may be rendered fit for the reception of the healthy: but I do not mean to inſiſt upon ſuch poſitions—it is treading upon very ſlippery ground—Dr. Trotter and myſelf are agreed as to the propriety of introducing oxygen gas into infectious wards; and if I have proved, as I truſt I have, that the nitric fumes have that effect, he will not, I hope, be ſo decidedly hoſtile to that proceſs. If Dr. Trotter does not know it, I now tell him, that I value his labours; I look up to his laudable activity with a pleaſurable anticipation of the reſult; and I heartily congratulate him on the important diſcuſſion which is now abroad, on a ſubject ſo intereſting to mankind.

To

* Medicina Nautica, Vol. II. p. 45.

